# Supplementary material for: Mechanisms governing the pioneering and redistribution capabilities of the non-classical pioneer PU.1
Source: Nat Commun. 2020 Jan 21;11:402. doi: 10.1038/s41467-019-13960-2 (PMC6972792; doi:10.1038/s41467-019-13960-2)
Supplement: Supplementary file 7 — Source data [file 41467_2019_13960_MOESM7_ESM.zip › Source_Data/FigureS2/FigureS2B_MotifScanOutput/KmeansCluster4/homerResults/motif4.similar.html]

motif4

## Information for motif4

A
G
T
C
C
G
T
A
T
A
C
G
C
G
A
T
A
C
G
T
A
G
T
C
A
G
C
T
T
G
A
C
  
Reverse Opposite:  

A
C
T
G
T
C
G
A
C
T
A
G
C
G
T
A
G
C
T
A
A
T
G
C
C
G
A
T
T
C
A
G
  

|  |  |
| --- | --- |
| p-value: | 1e-327 |
| log p-value: | -7.552e+02 |
| Information Content per bp: | 1.726 |
| Number of Target Sequences with motif | 1403.0 |
| Percentage of Target Sequences with motif | 31.07% |
| Number of Background Sequences with motif | 3762.5 |
| Percentage of Background Sequences with motif | 9.00% |
| Average Position of motif in Targets | 103.4 +/- 47.3bp |
| Average Position of motif in Background | 101.1 +/- 74.1bp |
| Strand Bias (log2 ratio + to - strand density) | 0.0 |
| Multiplicity (# of sites on avg that occur together) | 1.12 |
| Motif File: | file (matrix) reverse opposite |

### Similar de novo motifs found

|  |  |  |  |  |  |  |  |
| --- | --- | --- | --- | --- | --- | --- | --- |
| Rank | Match Score | Redundant Motif | P-value | log P-value | % of Targets | % of Background | Motif file |
| 1 | 0.879 | C G T A A C T G C T G A A C T G C G T A C G T A A T C G G A C T C T A G T A C G A G T C | 1e-194 | -446.849685 | 24.03% | 8.26% | motif file (matrix) |
| 2 | 0.887 | A G T C C G T A A T G C A C G T A C G T A G T C A G C T A T G C C G A T | 1e-165 | -380.256079 | 24.81% | 9.68% | motif file (matrix) |
